# Supplementary material for: Constitution of a comprehensive phytochemical profile and network pharmacology based investigation to decipher molecular mechanisms of Teucrium polium L. in the treatment of type 2 diabetes mellitus
Source: PeerJ. 2020 Oct 22;8:e10111. doi: 10.7717/peerj.10111 (PMC7585722; doi:10.7717/peerj.10111)
Supplement: Supplemental Information 4 [file peerj-08-10111-s004.docx]

| **Table S4.** The list of 126 compounds that were reported from *Teucrium polium* L. | | | | |
| --- | --- | --- | --- | --- |
| **No** | **Compound Group** | | **Compound Name** | **References** |
| **1** | Flavonoid | Phenolics | 4',7-dimethoxy apigenin | (Sharififar et al. 2008; Verykokidouvitsaropoulou & Vajias 1986; Esmaeili 2014) |
| **2** | Flavonoid | Phenolics | 4'-*O*-methyl luteolin (Diosmetin) | (Harborne et al. 1986; Mitreski et al. 2014; Stefkov et al. 2009; Venditti et al. 2017b; Tepe et al. 2011) |
| **3** | Flavonoid | Phenolics | 6-hydroxy luteolin | (Elmasri et al. 2015a) |
| **4** | Flavonoid | Phenolics | Acacetin | (Venditti et al. 2017a) |
| **5** | Flavonoid | Phenolics | Apigenin | (D'Abrosca et al. 2013; Esmaeili & Sadeghi 2009; Mitreski et al. 2014; Sharififar et al. 2008; Stefkov et al. 2009; Venditti et al. 2017a; Esmaeili 2014; Pacifico et al. 2012; Goulas et al. 2012) |
| **6** | Flavonoid | Phenolics | Catechin | ( Milosevic-Djordjevic et al. 2018) |
| **7** | Flavonoid | Phenolics | Cirsilineol | (Harborne et al. 1986; Stefkov et al. 2009; Venditti et al. 2017b) |
| **8** | Flavonoid | Phenolics | Cirsiliol | (Harborne et al. 1986; Mitreski et al. 2014; Stefkov et al. 2009; Venditti et al. 2017b; Verykokidouvitsaropoulou & Vajias 1986) |
| **9** | Flavonoid | Phenolics | Cirsimaritin | (Harborne et al. 1986; Kawashty et al. 1999; Mitreski et al. 2014; Stefkov et al. 2009; Venditti et al. 2017a; Venditti et al. 2017b; Verykokidouvitsaropoulou & Vajias 1986; Elmasri et al. 2016a) |
| **10** | Flavonoid | Phenolics | Eupatorin | (Verykokidouvitsaropoulou & Vajias 1986) |
| **11** | Flavonoid | Phenolics | Isoscutellarein | (Harborne et al. 1986) |
| **12** | Flavonoid | Phenolics | Jaceosidin (3',6-dimethoxy apigenin) | (Sharififar et al. 2008; Esmaeili 2014) |
| **13** | Flavonoid | Phenolics | Luteolin | (D'Abrosca et al. 2013; Harborne et al. 1986; Mitreski et al. 2014; Proestos et al. 2006; Stefkov et al. 2009; Venditti et al. 2017a; Pacifico et al. 2012; Tepe et al. 2011) |
| **14** | Flavonoid | Phenolics | Myricetin | (Milosevic-Djordjevic et al. 2018) |
| **15** | Flavonoid | Phenolics | Quercetin | (Harborne et al. 1986) |
| **16** | Flavonoid glycoside | Phenolics | Apigenin-7*-O*-glucoside (Cosmosiin) | (Kawashty et al. 1999; Mitreski et al. 2014; Venditti et al. 2017a; Goulas et al. 2012) |
| **17** | Flavonoid glycoside | Phenolics | Apigenin-7-*O*-rutinoside (Isorhoifolin) | (Mitreski et al. 2014; Venditti et al. 2017b; Goulas et al. 2012) |
| **18** | Flavonoid glycoside | Phenolics | Diosmetin-7-rutinoside (diosmin) | (Harborne et al. 1986; Mitreski et al. 2014) |
| **19** | Flavonoid glycoside | Phenolics | Kaempferol 7-*O*-diglucoside | (Mitreski et al. 2014) |
| **20** | Flavonoid glycoside | Phenolics | Luteolin 7-*O-β*-D-(5-*O*-syringyl)apiofuranosyl-(1 ⃗2)-*O-β*-D-glucopyranoside | (D'Abrosca et al. 2013) |
| **21** | Flavonoid glycoside | Phenolics | Luteolin 7-sambubioside | (Harborne et al. 1986) |
| **22** | Flavonoid glycoside | Phenolics | Luteolin-4'-*O*-glucoside | (De Marino et al. 2012) |
| **23** | Flavonoid glycoside | Phenolics | Luteolin-7-*O*-glucoside (Cynaroside) | (D'Abrosca et al. 2013; De Marino et al. 2012; Harborne et al. 1986; Kawashty et al. 1999; Mitreski et al. 2014; Tepe et al. 2011) |
| **24** | Flavonoid glycoside | Phenolics | Luteolin-7-*O*-rutinoside (Scolymoside) | (D'Abrosca et al. 2013; De Marino et al. 2012; Harborne et al. 1986; Mitreski et al. 2014; Tepe et al. 2011) |
| **25** | Flavonoid glycoside | Phenolics | Quercetin 3-glucoside (Isoquercitrin) | (Harborne et al. 1986) |
| **26** | Flavonoid glycoside | Phenolics | Rutin | (Esmaeili & Sadeghi 2009; Esmaeili 2014) |
| **27** | Flavonoid glycoside | Phenolics | Vicenin-2 | (Harborne et al. 1986; Kawashty et al. 1999) |
| **28** | Lignin | Phenolics | (7S,8R)-4-(*O*-*β*-D-glucopyranosyl)-dehydrodiconiferyl alcohol | (Elmasri et al. 2015a) |
| **29** | Lignin | Phenolics | (7S,8R)-5-methoxy-4-(*O*-*β*-D-glucopyranosyl)dehydrodiconiferyl alcohol (scorzonoside) | (Elmasri et al. 2015a) |
| **30** | Phenolic acid | Phenolics | Caffeic acid | (Proestos et al. 2006; Tepe et al. 2011) |
| **31** | Phenolic acid | Phenolics | Chlorogenic acid | (Milosevic-Djordjevic et al. 2018) |
| **32** | Phenolic acid | Phenolics | Gallic acid | (Milosevic-Djordjevic et al. 2018) |
| **33** | Phenolic acid | Phenolics | *p-*coumaric acid | (Milosevic-Djordjevic et al. 2018) |
| **34** | Phenolic acid | Phenolics | *t-*ferulic acid | (Proestos et al. 2006) |
| **35** | Phenolic acid | Phenolics | Vanillic acid | (Milosevic-Djordjevic et al. 2018) |
| **36** | Phenylethanol | Phenolics | 2-(3,4-dihydroxyphenyl)ethanol (hydroxytrosol) | (Elmasri et al. 2015a) |
| **37** | Phenylethanol | Phenolics | Tyrosol | (Proestos et al. 2006) |
| **38** | Phenylethanoid glycoside | Phenolics | 3-(*O*-*β*-D-glucopyranosyl)-*α*-(*O-β*-D-glucopyranosyl)-4-hydroxyphenylethanol | (Elmasri et al. 2015a) |
| **39** | Phenylethanoid glycoside | Phenolics | 3,4-dihydroxy-3-(*O-β*-D-glucopyranosyl)phenethanol | (Elmasri et al. 2015a) |
| **40** | Phenylethanoid glycoside | Phenolics | Allysonoside | (Mitreski et al. 2014) |
| **41** | Phenylethanoid glycoside | Phenolics | Echinacoside | (Mitreski et al. 2014) |
| **42** | Phenylethanoid glycoside | Phenolics | Forsythoside A (Forsythiaside) | (Mitreski et al. 2014) |
| **43** | Phenylethanoid glycoside | Phenolics | Forsythoside B | (Mitreski et al. 2014) |
| **44** | Phenylethanoid glycoside | Phenolics | Leucosceptoside A | (Mitreski et al. 2014) |
| **45** | Phenylethanoid glycoside | Phenolics | Poliumoside | (De Marino et al. 2012; Oganesyan et al. 1991; Venditti et al. 2017b; Elmasri et al. 2016a; Elmasri et al. 2014; Pacifico et al. 2012; Goulas et al. 2012) |
| **46** | Phenylethanoid glycoside | Phenolics | Poliumoside B | (De Marino et al. 2012; Mitreski et al. 2014) |
| **47** | Phenylethanoid glycoside | Phenolics | Samioside | (Mitreski et al. 2014) |
| **48** | Phenylethanoid glycoside | Phenolics | Teupolioside | (Oganesyan et al. 1991) |
| **49** | Phenylethanoid glycoside | Phenolics | Acteoside | (Mitreski et al. 2014; Oganesyan et al. 1991; Venditti et al. 2017a; Elmasri et al. 2016a; Goulas et al. 2012; Tepe et al. 2011) |
| **50** | Secoiridoid | Terpenoids | 4-[(*β*-D-glucopyranosyloxy)methylene]-5*α*-(2-hydroxyethyl)-5-(*α*-L-rhamnopyranosyloxy)-3-methylcyclopent-2-en-1-one | (Elmasri et al. 2015a) |
| **51** | Secoiridoid | Terpenoids | 4*α*-[(*β*-D-glucopyranosyloxy)methyl]-5*α*-(2-hydroxyethyl)-3-methylcyclopent-2-en-1-one | (Elmasri et al. 2015a; Elmasri et al. 2016b) |
| **52** | Secoiridoid | Terpenoids | 5*α*-[2-(*β*-D-glucopyranosyloxy)ethyl]-4*α*-hydroxymethyl-3-methylcyclopent-2-en-1-one | (Elmasri et al. 2015a; Elmasri et al. 2016b) |
| **53** | Secoiridoid | Terpenoids | 5*α*-(2-hydroxyethyl)-4*α*-hydroxymethyl-3-methylcyclopent-2-en-1-one | (Elmasri et al. 2015a) |
| **54** | Iridoid glycoside | Terpenoids | 8-*O*-acetyl harpagide | (De Marino et al. 2012; Elmasri et al. 2016a) |
| **55** | Iridoid glycoside | Terpenoids | Teucardoside | (De Marino et al. 2012; Elmasri et al. 2016a; Elmasri et al. 2016b Elmasri et al. 2014) |
| **56** | Iridoid glycoside | Terpenoids | Teuhircoside | (Elmasri et al. 2015a; Elmasri et al. 2016b) |
| **57** | Iridoid glycoside | Terpenoids | 1*α*-(*β*-D-glucopyranoxy)-6*α*,7*α*-epoxy-4aβ,5α-dihydroxy-7-methyl-1,4a,5,6,7,7aβ-hexahydrocyclopenta[c]pyran | (Elmasri et al. 2015a) |
| **58** | Diterpenoid | Terpenoids | 10-*β*-hydroxy-teucjaponin B | (Venditti et al. 2017b) |
| **59** | Diterpenoid | Terpenoids | 19-deacetylteuscorodol | (Fiorentino et al. 2011) |
| **60** | Diterpenoid | Terpenoids | 20-*O*-acetyl-teucrasiatin | (Venditti et al. 2017a) |
| **61** | Diterpenoid | Terpenoids | 7-hydroxy-6-ketone picropolin | (Marquez & Valverde 1979) |
| **62** | Diterpenoid | Terpenoids | Capitatin | (Malakov & Papanov 1983; Bruno et al. 2003) |
| **63** | Diterpenoid | Terpenoids | Clerodane-6,7-dione | (Marquez & Valverde 1979) |
| **64** | Diterpenoid | Terpenoids | Montanin B | (Fiorentino et al. 2011) |
| **65** | Diterpenoid | Terpenoids | Montanin D | (Fiorentino et al. 2011; Pacifico et al. 2012) |
| **66** | Diterpenoid | Terpenoids | Montanin E | (Fiorentino et al. 2011; Pacifico et al. 2012) |
| **67** | Diterpenoid | Terpenoids | Phytol | (Venditti et al. 2017a) |
| **68** | Diterpenoid | Terpenoids | Picropolin | (Venditti et al. 2017b) |
| **69** | Diterpenoid | Terpenoids | Teubutilin A | (Fiorentino et al. 2011; Pacifico et al. 2012) |
| **70** | Diterpenoid | Terpenoids | Teuchamaecrin C | (Fiorentino et al. 2011; Pacifico et al. 2012) |
| **71** | Diterpenoid | Terpenoids | Teucrasiatin | (Venditti et al. 2017a) |
| **72** | Diterpenoid | Terpenoids | Teucroxylepin | (Fiorentino et al. 2011) |
| **73** | Diterpenoid | Terpenoids | Teukotschyn | (Fiorentino et al. 2011) |
| **74** | Diterpenoid | Terpenoids | Teulamifin B | (De Marino et al. 2012; Fiorentino et al. 2011; Pacifico et al. 2012) |
| **75** | Diterpenoid | Terpenoids | Teulolin A | (Bedir et al. 1999) |
| **76** | Diterpenoid | Terpenoids | Teulolin B | (Bedir et al. 1999) |
| **77** | Diterpenoid | Terpenoids | Teupolin I | (Venditti et al. 2017b) |
| **78** | Diterpenoid | Terpenoids | Teupolin IV | (Malakov & Papanov 1983) |
| **79** | Diterpenoid | Terpenoids | Teupolin IX | (Fiorentino et al. 2011; Pacifico et al. 2012) |
| **80** | Diterpenoid | Terpenoids | Teupolin V | (Malakov & Papanov 1983) |
| **81** | Diterpenoid | Terpenoids | Teupolin VI | (Fiorentino et al. 2011; Pacifico et al. 2012) |
| **82** | Diterpenoid | Terpenoids | Teupolin VII | (Fiorentino et al. 2011; Pacifico et al. 2012) |
| **83** | Diterpenoid | Terpenoids | Teupolin VIII | (Fiorentino et al. 2011; Pacifico et al. 2012) |
| **84** | Diterpenoid | Terpenoids | Teupolin X | (Fiorentino et al. 2011; Pacifico et al. 2012) |
| **85** | Diterpenoid | Terpenoids | Teupolin XI | (Fiorentino et al. 2011; Pacifico et al. 2012) |
| **86** | Diterpenoid | Terpenoids | Teupolin XII | (Fiorentino et al. 2011; Pacifico et al. 2012) |
| **87** | Diterpenoid | Terpenoids | Teusalvin C | (De Marino et al. 2012; Fiorentino et al. 2011) |
| **88** | Triterpenoid | Terpenoids | Maslinic acid | (Venditti et al. 2017a) |
| **89** | Triterpenoid | Terpenoids | Oleanolic acid | (Venditti et al. 2017a) |
| **90** | Nonsesquiterpenoid | Terpenoids | (1R, 4S, 10R) 10,11-dimethyl-dicyclohex-5(6)-en-1,4-diol-7-one | (Elmasri et al. 2016a) |
| **91** | Cyanogenic glycoside | Amino acid derivative | (R)-mandelonitrile-β-laminaribioside | (Elmasri et al. 2016a) |
| **92** | Sesquiterpenoid | Terpenoids | (1R,6R,7R,8S,11R)-1,6-dihydroxy-4,11-dimethyl-germacran-4(5), 10(14)-dien-8,12-olide | (Elmasri et al. 2016a) |
| **93** | Sesquiterpenoid | Terpenoids | (10R,1R,4S,5S,6R,7S)-4,10-die-poxygermacran-6-ol | (Elmasri et al. 2016a) |
| **94** | Phenylethanoid glycoside | Phenolics | 2-(3-hydroxy-4-methoxyphenyl)-ethyl-O-(αL-rhamnosyl)-(1->3)-O-(α-L-rhamnosyl)-(1->6)-4-O-E-feruloyl-β-D-glucopyranoside | (Elmasri et al. 2016a) |
| **95** | Cyanogenic glycoside | Amino acid derivative | Prunasin | (Elmasri et al. 2016a) |
| **96** | Nonsesquiterpenoid | Terpenoids | 1α-hydroxy isoondetamnone | (Elmasri et al. 2016a) |
| **97** | Saponin | Terpenoids | Poliusaposide A | (Elmasri et al. 2015b) |
| **98** | Saponin | Terpenoids | Poliusaposide B | (Elmasri et al. 2015b) |
| **99** | Saponin | Terpenoids | Poliusaposide C | (Elmasri et al. 2015b) |
| **100** | Sesquiterpenoid | Terpenoids | 4β,5α-Epoxy-7αH-germacr-10(14)-en-6β-ol-1-one | (Elmasri et al. 2014) |
| **101** | Sesquiterpenoid | Terpenoids | 4β,5α-Epoxy-7αH-germacr-10(14)-en,1β-hydroperoxyl,6β-ol | (Elmasri et al. 2014) |
| **102** | Sesquiterpenoid | Terpenoids | 4β,5β-Epoxy-7αH-germacr-10(14)-en,1β-hydroperoxyl,6β-ol | (Elmasri et al. 2014) |
| **103** | Sesquiterpenoid | Terpenoids | 4α,5β-epoxy-7αH-germacr-10(14)-en,1β-hydroperoxyl,6α-ol | (Elmasri et al. 2014) |
| **104** | Sesquiterpenoid | Terpenoids | 10α,1β;4β,5α-diepoxy-7αH-germacrm-6-ol | (Elmasri et al. 2014) |
| **105** | Sesquiterpenoid | Terpenoids | Teucladiol | (Elmasri et al. 2014) |
| **106** | Sesquiterpenoid | Terpenoids | 4β,6β-dihydroxy-1α,5β(H)-guai-9-ene | (Elmasri et al. 2014) |
| **107** | Sesquiterpenoid | Terpenoids | Oplopanone | (Elmasri et al. 2014) |
| **108** | Sesquiterpenoid | Terpenoids | Oxyphyllenodiol A | (Elmasri et al. 2014) |
| **109** | Sesquiterpenoid | Terpenoids | rel-1β,3α,6β-trihydroxyeudesm-4-ene | (Elmasri et al. 2014) |
| **110** | Sesquiterpenoid | Terpenoids | Arteincultone | (Elmasri et al. 2014) |
| **111** | Flavonoid | Phenolics | 5,6-Dihydroxy-7,4'-dimethoxyflavone (Ladanein) | (Elmasri et al. 2014) |
| **112** | Flavonoid | Phenolics | Salvigenin | (Elmasri et al. 2014) |
| **113** | Iridoid glycoside | Terpenoids | 1α-(β-D-glucopyranoxy)-7α,8α-epoxy-5β,6α-dihydroxy-8-methyl-1,5,6,7,8,9β-hexahydrocyclopenta[c]pyran | (Elmasri et al. 2016b) |
| **114** | Iridoid glycoside | Terpenoids | (4aS)‐7‐methyl‐1‐{[(3R,4S,5S,6R)‐3,4,5‐trihydroxy‐6‐  (hydroxymethyl)oxan‐2‐yl]oxy}‐4a‐{[(3S,4S,5S,6R)‐  3,4,5‐trihydroxy‐6‐methyloxan‐2‐yl]oxy}‐  3H,4H,4aH,5H‐cyclopenta[c]pyran‐5‐one | (Elmasri et al. 2016b) |
| **115** | Flavonoid glycoside | Phenolics | Apigenin 4'-O-glucoside | (Goulas et al. 2012) |
| **116** | Flavonoid | Phenolics | 5,3',4'- trihydroxy-3,7-dimethoxyflavone | (Goulas et al. 2012) |
| **117** | Flavonoid | Phenolics | Kumatakenin (Jaranol) | (Goulas et al. 2012) |
| **118** | Flavonoid glycoside | Phenolics | Diosmetin 7-O-glucoside | (Tepe et al. 2011) |
| **119** | Flavonoid glycoside | Phenolics | Apigenin 7-O-glucuronide | (Tepe et al. 2011) |
| **120** | Diterpenoid | Terpenoids | Auropolin | (Bruno et al. 2003) |
| **121** | Diterpenoid | Terpenoids | 20-epi-auropolin | (Bruno et al. 2003) |
| **122** | Sesquiterpenoid | Terpenoids | β-eudesmol | (Alaa 1995) |
| **123** | Sesquiterpenoid | Terpenoids | α-Cadinol | (Alaa 1995) |
| **124** | Sesquiterpenoid | Terpenoids | 7-epi-Eudesm-4(15)-ene-lβ,6α-diol | (Alaa 1995) |
| **125** | Sesquiterpenoid | Terpenoids | 7-epi-Eudesm-4(15)-ene-lβ,6β-diol | (Alaa 1995) |
| **126** | Diterpenoid | Terpenoids | Teupolin III | (Malakov et al. 1982) |

Alaa K. 1995. 7-Epi-Eudesmanes from *Teucrium polium*. *Journal of Natural Products* 58:428-431.

Bedir E, Tasdemir D, Calis I, Zerbe O, and Sticher O. 1999. Neo-clerodane diterpenoids from *Teucrium polium*. *Phytochemistry* 51:921-925. DOI:10.1016/S0031-9422(99)00052-7

Bruno M, Maggio AM, Piozzi F, Puech S, Rosselli S, Simmonds SJ. 2003. Neoclerodane diterpenoids from *Teucrium polium* subsp. *polium* and their antifeedant activity. *Biochemical Systematic and Ecology* 31:1051-156. DOI: 10.1016/S0305-1978(03)00042-5

D'Abrosca B, Pacifico S, Scognamiglio M, D'Angelo G, Galasso S, Monaco P, and Fiorentino A. 2013. A new acylated flavone glycoside with antioxidant and radical scavenging activities from *Teucrium polium* leaves. *Natural Product Research* 27:356-363. DOI:10.1080/14786419.2012.695367

De Marino S, Festa C, Zollo F, Incollingo F, Raimo G, Evangelista G, and Iorizzi M. 2012. Antioxidant activity of phenolic and phenylethanoid glycosides from *Teucrium polium* L. *Food Chemistry* 133:21-28. DOI:10.1016/j.foodchem.2011.12.054

Elmasri WA, Hegazy ME, Aziz M, Koksal E, Amor W, Mechref Y, Hamood AN, Cordes DB and Pare PW. 2014. Biofilm blocking sesquiterpenes from *Teucrium polium*. *Phytochemistry* 103:107-113. DOI: 10.1016/j.phytochem.2014.03.029

Elmasri WA, Hegazy ME, Mechref Y, and Pare PW. 2015b. Cytotoxic saponin poliusaposide from *Teucrium polium*. *RSC Advances* 5:27126. DOI: 10.1039/c5ra02713f

Elmasri WA, Hegazy ME, Mechref Y, and Pare PW. 2016a. Structure-antioxidant and anti-tumor activity of *Teucrium polium* phytochemicals. *Phytochemistry Letters* 15:81-87. DOI:10.1016/j.phytol.2015.11.007

Elmasri WA, Yang T, Tran P, Hegazy ME, Hamood AN, Mechref Y, and Pare PW. 2015a. *Teucrium polium* phenylethanol and iridoid glycoside characterization and flavonoid inhibition of biofilm-forming Staphylococcus aureus. *Journal of Natural Products* 78:2-9. DOI:10.1021/np5004092

Elmasri WA, Yang T, Hegazy ME, Mechref Y, and Pare PW. 2016b. Iridoid glycoside permethylation enhances chromatographic separation and chemical ionization. *Rapid Communications Mass Spectrometry* 30:2033-2042. DOI: 10.1002/rcm.7681

Esmaeili MA. 2014. Advanced Glycation End Product Inhibitory Effect of Quercetin-3-O-Rutinoside, Isolated from *Teucrium polium*. *Pharmaceutical sciences* 19:125-134.

Esmaeili MA, and Sadeghi H. 2009. Pancreatic β-cell protective effect of rutin and apigenin isolated from *Teucrium polium*. *Pharmacologyonline* 2:341-353.

Fiorentino A, D'Abrosca B, Pacifico S, Scognamiglio M, D'Angelo G, Gallicchio M, Chambery A, and Monaco P. 2011. Structure elucidation and hepatotoxicity evaluation against HepG2 human cells of neo-clerodane diterpenes from *Teucrium polium* L. *Phytochemistry* 72:2037-2044. DOI:10.1016/j.phytochem.2011.07.006

Goulas V, Gomez-Caravaca AM, Exarchou V, Gerothanassis IP, Segura-Carretero A, Gutierrez AF. 2012. Exploring the antioxidant potential of *Teucrium polium* extracts by HPLC-SPE-NMR and on-line radical-scavenging activity detection. *LWT-Food Science and Technology* 46:104-109. DOI: 10.1016/j.lwt.2011.10.019

Harborne JB, Tomasbarberan FA, Williams CA, and Gil MI. 1986. A chemotaxonomic study of flavonoids from european Teucrium species. *Phytochemistry* 25:2811-2816. DOI:10.1016/S0031-9422(00)83747-4

Kawashty SA, El-Din EMG, and Saleh NAM. 1999. The flavonoid chemosystematics of two Teucrium species from Southern Sinai, Egypt. *Biochemical Systematics and Ecology* 27:657-660. DOI:10.1016/S0305-1978(97)00109-9

Malakov PY, and Papanov GY. 1983. Furanoid Diterpenes from *Teucrium polium*. *Phytochemistry* 22:2791-279. <https://doi.org/10.1016/S0031-9422(00)97698-2>

Malakov PY, Papanov GY and Ziesche J. 1982. Teupolin III, a Furanoid Diterpene from *Teucrium polium*. Phytochemistry 21: 2597-2598.

Marquez C, and Valverde S. 1979. A new clerodane diterpene from *Teucrium polium* L. *Journal of the Chemical Society*, *Pekin Transactions* 1:2526-2527. DOI: 10.1039/p19790002526

Milosevic-Djordjevic O, Radovic Jakovljevic M, Markovic A, Stankovic M, Ciric A, Marinkovic D, and Grujicic D. 2018. Polyphenolic contents of *Teucrium polium* L. and *Teucrium scordium* L. associated with their protective effects against MMC-induced chromosomal damage in cultured human peripheral blood lymphocytes. *Turkish Journal of Biology* 42:152-162. DOI:10.3906/biy-1707-36

Mitreski I, Stanoeva JP, Stefova M, Stefkov G, and Kulevanova S. 2014. Polyphenols in Representative *Teucrium* species in the Flora of R. Macedonia: LC/DAD/ESI-MSn profile and content. *Natural Product Communications* 9:175-180. DOI: 10.1177/1934578x1400900211

Oganesyan GB, Galstyan AM, Mnatsakanyan VA, Shashkov AS, and Agababyan RV. 1991. phenylpropanoid glycosides of *Teucrium polium*. *Khimiya Prirodnykh Soedinenii*:630-634. DOI: 10.1007/bf00630353

Pacifico S, D’Abrosca B, Scognamiglio M, D’Angelo, Gallicchio M, Galasso S, Monaco P, Fiorentino A. 2012. NMR-based metabolic profiling and in vitro antioxidant and hepatotoxic assessment of partially purified fractions from Golden germander (*Teucrium polium* L.) methanolic extract. *Food Chemistry* 135: 1957-1967. DOI: 10.1016/j.foodchem.2012.06.071

Proestos C, Sereli D, and Komaitis M. 2006. Determination of phenolic compounds in aromatic plants by RP-HPLC and GC-MS. *Food Chemistry* 95:44-52. DOI:10.1016/j.foodchem.2004.12.016

Sharififar F, Dehghn-Nudeh G, and Mirtajaldini M. 2008. Major flavonoids with antioxidant activity from *Teucrium polium* L. *Food Chemistry* 112:885-888. DOI:10.1016/j.foodchem.2008.06.064

Stefkov G, Karapandzova M, Stefova M, and Kulevanova S. 2009. Seasonal variation of flavonoids in *Teucrium polium* L. (Lamiaceae). *Macedonian pharmaceutical bulletin* 55:33-40. DOI: 10.33320/maced.pharm.bull.2009.55.003

Tepe B, Degerli S, Arslan S, Malatyali E, Sarikurkcu C. 2011. Determination of chemical profile, antioxidant, DNA damage protection and antiamoebic activities of *Teucrium polium* and *Stachys iberica* . *Fitoterapia* 82:237-246. DOI: 10.1016/j.fitote.2010.10.006

Venditti A, Frezza C, Trancanella E, Zadeh SMM, Foddai S, Sciubba F, Delfini M, Serafini M, and Bianco A. 2017a. A new natural neo-clerodane from *Teucrium polium* L. collected in Northern Iran. *Industrial Crops and Products* 97:632-638. DOI:10.1016/j.indcrop.2017.01.010

Venditti A, Frezza C, Zadeh SMM, Foddai S, Serafini M, and Bianco A. 2017b. Secondary metabolites from *Teucrium polium* L. collected in Southern Iran. *Arabian Journal of Medicinal & Aromatic Plants* 3:108-123.

Verykokidouvitsaropoulou E, and Vajias C. 1986. Methylated flavones from *Teucrium polium*. *Planta Medica*:401-402. DOI: 10.1055/s-2007-969198
